# Another step closer to measuring the ghosts in the nursery: preliminary validation of the Trauma Reflective Functioning Scale

**DOI:** 10.3389/fpsyg.2014.01471

**Published:** 2014-12-17

**Authors:** Karin Ensink, Nicolas Berthelot, Odette Bernazzani, Lina Normandin, Peter Fonagy

**Affiliations:** ^1^Université LavalLaval, QC, Canada; ^2^Université du Québec à Trois-RivièresTrois-Rivières, QC, Canada; ^3^Université de MontréalMontréal, QC, Canada; ^4^Research Department of Clinical, Educational and Health Psychology, University College LondonLondon, UK; ^5^Anna Freud CentreLondon, UK

**Keywords:** reflective functioning, mentalization, trauma, unresolved trauma, attachment, childhood abuse and neglect, transition to motherhood

## Abstract

The aim of this study was to examine preliminary evidence of the validity of the Trauma Reflective Functioning Scale and to investigate reflective functioning (RF) and attachment in pregnant women with histories of trauma, with a particular focus on the capacity to mentalize regarding trauma and its implications for adaptation to pregnancy and couple functioning. The Adult Attachment Interview was used to assess attachment, unresolved trauma and mentalization (measured as RF) regarding relationships with attachment figures (RF-G) and trauma (RF-T) in 100 pregnant women with histories of abuse and neglect. The majority (63%) of women had insecure attachment states of mind and approximately half were unresolved regarding trauma. Furthermore, the majority of women manifested deficits specific to RF-T. Their RF-T was significantly lower than their RF-G; the findings indicate that women with histories of childhood abuse and neglect do not manifest a generic inhibition of reflectiveness, but a collapse of mentalization specific to trauma. Low RF-T, indicative of difficulty in considering traumatic experiences in mental state terms, was associated with difficulty in investment in the pregnancy and lack of positive feelings about the baby and motherhood. In addition, low RF-T was also associated with difficulties in intimate relationships. Results of a regression analysis with RF indicated that RF-T was the best predictor of investment in pregnancy and couple functioning. In sum, the study provides preliminary evidence that RF-T can be reliably measured and is a valid construct that has potential usefulness for research and clinical practice. It highlights the importance of mentalization specifically about trauma and suggests that it is not the experience of trauma *per se*, but the *absence of mentalization regarding trauma* that is associated with difficulties in close relationships and in making the transition to parenthood.

## INTRODUCTION

The transition to motherhood is recognized as being an important developmental period of psychic transformation and potential reworking of unresolved issues, such as childhood abuse or neglect (CA&N), that might have an impact on the future role of parent (Slade et al., 2009; Raphael-Leff, 2010; Ammaniti et al., 2013). Fraiberg et al. (1975) evocatively referred to these unresolved traumatic experiences as “ghosts in the nursery.” In spite of the growing body of research on the impact of abuse and neglect on infant attachment, there is surprisingly little research regarding the long-term implications of CA&N for *adult* attachment and reflective functioning (RF) to inform prospective parents and mental health professionals. Systematic knowledge of the implications of CA&N for adult attachment and mentalization would seem a matter of priority given that secure attachment and high RF are considered important protective factors in contexts of childhood deprivation and potential mediating mechanisms of the intergenerational impacts of childhood maltreatment (Fonagy et al., 1994; Kwako et al., 2010). Furthermore, despite extensive theoretical writings on the impact of CA&N on the development of mentalization capacities (Fonagy and Luyten, 2009; Allen, 2013), few empirical studies have examined this relationship.

Whereas Fraiberg et al. (1975) conceptualized “ghosts in the nursery” in terms of the voices of the past and a haunting presence left by trauma, Fonagy (1993) viewed the concept as an *absence of mentalization* about emotionally painful experiences of fear and helplessness that makes the parent vulnerable to identifying with the aggressor rather than responding to the distress of the infant. Initially, this absence of mentalization was conceptualized as a deficit in mentalization about attachment relationships. However, it remains important to clarify whether mentalization capacities about attachment relationships are compromised *globally* following CA&N or whether deficits in mentalization are *specific* to traumatic experiences, and, furthermore, whether deficits in mentalization in general, or deficits in mentalization regarding trauma in particular, are associated with difficulties in adaptation, parenting, and relationships. Fraiberg observed that what distinguishes those who make a conscious effort not to repeat past trauma has to do with being prepared to remember the pain of past trauma, and that this protects them from repeating the trauma. This would suggest that mentalization specific to trauma, rather than mentalization in general, is likely to be particularly important for parents who have histories of trauma and, by implication, for the intergenerational transmission of trauma.

### ATTACHMENT AND UNRESOLVED TRAUMA IN THE CONTEXT OF ABUSE

Given the close and complementary relationship between attachment and RF, it is important to consider the current state of knowledge with regard to attachment in adults with CA&N to understand the unique relationship between RF and trauma. There are few published studies addressing attachment in adults with histories of CA&N. A meta-analysis of research using the Adult Attachment Interview (AAI) conducted by Bakermans-Kranenburg and van IJzendoorn (2009) reported that only five studies examined attachment in adults with CA&N (Stalker and Davies, 1998; Ziegenhain et al., 2004; Riggs et al., 2007; Stovall-McClough et al., 2008; Taylor-Seehafer et al., 2008), accounting for 263 out of 10,000 AAIs. In addition, the majority of existing studies of attachment in the context of CA&N focused on high-risk or clinical samples; in such samples, 86% of individuals with histories of abuse had insecure attachment representations and the majority (68%) was unresolved regarding trauma. In the only study to include AAI data of adults with histories of abuse not recruited from clinical or high-risk samples, Stovall-McClough and Cloitre (2006) found lower rates of unresolved trauma and higher rates of secure attachment states of mind. They compared the attachment styles of 30 women with histories of abuse with or without posttraumatic stress disorder (PTSD); they found that, of the women without PTSD, 37% had secure attachment states of mind and 27% were unresolved regarding loss or trauma, compared with rates of 17% for secure attachment representations and 63% for unresolved loss or trauma for the group of women with PTSD. This suggests that the profiles of adults with CA&N might be more varied in terms of attachment security and resolution of trauma than previous studies with clinical and high-risk samples have led us to expect. For example, abuse can occur in the context of a “reign of terror” and severe discipline and emotional neglect by both parents, whereas other parents may love their children but become abusive in situations where repressed experiences from childhood are activated (Renk et al., 2004). Furthermore, a dose–response relationship between the number and severity of traumatic events and outcomes such as PTSD and anxiety (Brewin et al., 2000) has frequently been observed, but data in support of a similar dose–response relationship in relation to attachment are still lacking.

### REFLECTIVE FUNCTIONING

In a seminal study, Fonagy et al. (1994) found that mothers who had experienced risk and deprivation, but had high RF, were much more likely to have securely attached infants, suggesting that mentalization is a potential mediator of the intergenerational transmission of risk. As Allen (2013) points out, the research findings regarding the importance of parental mentalization for infant attachment can be considered a significant refinement of Ainsworth’s pioneering work on the importance of sensitive parenting. “Reflective functioning” and “mentalization” are used interchangeably and refer to the sociocognitive capacity to think about oneself and others as psychological beings and to consider underlying mental states and motivations when interpreting behaviors in attachment contexts (Choi-Kain and Gunderson, 2008). Considering the implications of RF for parenting, there is a need for further data on the impact of CA&N experiences on the mentalization capacity of adults.

Fonagy and Target (1997, 2006) emphasized the link between attachment and mentalization, as RF is developmentally rooted in attachment relationships in which children experience their mental states being reflected upon by a caregiver who is able to consider and respond to the emotional states of the child. This is theorized to create a space in which the child can discover about minds, including his/her own, and in which an understanding of mental and emotional life can develop (Bram and Gabbard, 2001). The experience of being perceived by the caregiver as someone with a mind is considered central to the development of the child’s capacity to create a coherent image of his/her own mind and self (Fonagy et al., 2007). As Fonagy and Target (2006) observed, it is difficult to imagine being able to develop the capacity to imagine the minds of others and be mentalizing in relation to others without the experience of having been treated as someone with a mind—so that, as Allen (2013) puts it, mentalization begets mentalization.

More recently, Fonagy and Luyten (2009) proposed that attachment and RF are *loosely* connected, because although mentalization originates in the context of attachment relationships, it subsequently has a distinct developmental path with specific factors contributing to its further development, such as family mental state talk and access to an adult who has an interest in the internal world of the child and who can help him/her mentalize difficult experiences. This is usually associated with secure attachment relationships, but it is possible that some individuals are able to benefit from other relationships to develop mentalization capacities even if their initial attachment was insecure.

While the general capacity to mentalize about attachment relationships is argued to have important implications for adaptive functioning, strengths and weaknesses in particular mentalization capacities are likely to be the best predictors of adaptive functioning in different domains such as parenting and mental health (Rudden et al., 2009; Luyten et al., 2012). In line with this conceptualization, in the context of CA&N, RF regarding *trauma* may be expected to be particularly challenging and yet also particularly important for adaptive functioning, the regulation of affects such as fear, anger, and vulnerability, and the ability to confide and have trust in others in intimate relationships.

### REFLECTIVE FUNCTIONING AND RESILIENCE IN THE CONTEXT OF ABUSE

From a developmental perspective, and considering the importance of the parent’s reflective stance for parent–child interactions through which children learn about themselves and others and develop their mentalization capacities, it is not surprising that deficits across a range of mentalization capacities have been identified in maltreated children. These deficits include poor mentalization capacities (Ensink et al., 2014) poor discrimination of emotions (Pollak et al., 2000; Edwards et al., 2005), theory of mind (Cicchetti et al., 2003; Pears and Fisher, 2005), and emotional understanding (Rogosch et al., 1995; Shipman and Zeman, 1999). There is evidence that maltreating parents engage their children less often in emotional discussions (Edwards et al., 2005) and that they manifest impairments in their capacity to understand their children’s expression of affect (Shipman and Zeman, 2001). Maltreating parents may thus be unable or unwilling to establish the type of relationships and interactions in which the child can learn about their own and others’ minds. This may be because maltreating parents themselves have difficulties in mentalizing and are unable or unwilling to adopt a mentalizing stance toward their child and to imagine the child’s internal experience. Furthermore, they may discourage coherent discourse about mental states and undermine the development of mentalization in the child, to avoid engaging with the psychological impact and suffering they inflict on the child (Allen et al., 2008; Fonagy and Luyten, 2009).

In addition to the negative impact CA&N is expected to have on the development of mentalization capacities in general, adults with CA&N are likely to manifest even more profound difficulties in mentalizing regarding trauma. First, the complex and confusing psychological experiences and reactions in the context of abuse are likely to be particularly challenging to mentalize (Cloitre et al., 2011). As Perry (2009) observed, because traumatic events have features that are so far outside normal experience, it is very difficult to use what has been learned from ordinary experience to judge and make sense of such events. On their own, children are unlikely to succeed in elaborating a verbal and mentalized account of traumatic experiences unless they have recourse to a trusted adult in whom they can confide and who is motivated to try to understand and imagine their experience and help them elaborate a narrative about what happened (Ensink et al., 2014). Without such narratives, where experience is represented verbally so that memories can become explicit (declarative–verbal), traumatic memories are likely to remain implicit (non-declarative, perceptual–motor) and close to the primary experience (Brewin, 2011). This would account for why, when these implicit memories of trauma are triggered, certain aspects of the trauma are relived as if they were happening in the present, and overwhelming feeling states related to the trauma are re-experienced (Allen, 2013). Third, to preserve their attachment relationships, children may be too terrified to think about the abuse and the minds of caregivers who sometimes harbor malevolent intentions or have distorted representations of them (Fonagy and Target, 2006; Allen, 2013). Finally, children in attachment relationships with abusive parents frequently resort to dissociation (Briere, 2002; Berthelot et al., 2012). This facilitates the re-establishment of regulation and adaptive functioning by compartmentalizing trauma related memories (Steele and Van der Hart, 2009), but at the cost of inhibiting mentalization (Allen, 2013).

For the child who lives with an abusive attachment figure who is likely to become the source of fear rather than safety, permanent activation of the attachment system, hypervigilance, and scanning the environment and parents’ facial expressions for possible threats are commonly documented adaptations (Cicchetti and Toth, 2005; De Bellis, 2005). Fonagy and Luyten (2009) proposed that a dual process model (Arnsten, 1998; Mayes, 2006; Lieberman, 2007) is particularly relevant for understanding the impact of attachment trauma, with children with abusive attachment figures being more likely to predominantly use an automatic reflexive mode (Lieberman, 2007) adapted to detect threat, rather than the slower reflective mode involving the prefrontal cortex. In the reflexive mode, where the focus is on the avoidance of threat, a more primitive neurobiological system developed for the fast processing of interpersonal and social information is predominantly activated. This mode is well adapted for rapid analysis of external signs and behaviors that might signal danger, and relies on past experiences so that fast decisions can be made. Arnsten (1998) points out the irony that, even in individuals who are usually highly reflective and rational, at a certain level of stress there is a biological switch to the reflexive mode, so that reflective capacity is lost when it is most needed. The problem of maintaining the capacity to be reflective when under stress is likely to be compounded for individuals who grew up in contexts of potential threat, who are expected to have predominantly used reflexive rather than reflective modes. For these reasons, mentalization regarding trauma is likely to be important for maintaining reflective thinking in interpersonal contexts when trauma-related affects are triggered.

### INVESTMENT IN PREGNANCY IN MOTHERS WITH A HISTORY OF CHILDHOOD ABUSE AND NEGLECT

Pregnancy is considered to be a crucial transitional period in a woman’s life, and an adequate negotiation of what Slade et al. (2009) refer to as the *developmental crisis of pregnancy* is essential for the future mental health of both the mother and the baby. Pregnancy entails an important psychological transformation whereby the pregnant woman has to reorganize her identity as a woman to include her identity not just as a mother, but also as a caregiver (Slade et al., 2009; Raphael-Leff, 2010; Ammaniti et al., 2013). This is inevitably closely connected to her own history and childhood experiences (Fraiberg et al., 1975) and reactivates her representations of her own mother, and involves a deep reworking of her representation of being a mother as part of the psychic reorganization that Stern (1995) refers to as the “motherhood constellation.” This reorganization is likely to be fraught if her relationships with her own mother and caregivers have been complex and traumatic. Women with past experiences of CA&N can be expected to find it more difficult to make the psychological shift to becoming the provider of care and protection (George and Solomon, 2008), and it is arguably more challenging for women to feel psychologically prepared to provide care and protection when their own needs for care and protection were not met (Huth-Bocks et al., 2013). At the same time as reworking her internal representations of parenting and assimilating her identity as a mother, the pregnant woman also has to develop an attachment to the future baby and activate the caregiving system (Winnicott, 1969; Ammaniti et al., 2014), which is considered to be a primary motivational system that competes with other motivational systems such as pair-bonding and sexuality.

With regard to RF, pregnant women who are able to think about early attachment relationships that have been difficult in mental state terms and who are able to mentalize about traumatic experiences can be expected to find the transition to motherhood less fraught. Following Fonagy’s (1993) proposal that mentalization has a crucial protective role in the context of “ghosts in the nursery,” we expected that pregnant women who were able to think about trauma and who had been able to develop an account of traumatic experiences in mental state terms would be more able to invest in their pregnancy, experience positive affects about the baby and motherhood, and experience fewer negative feelings about the pregnancy.

### THE QUALITY OF COUPLE RELATIONSHIPS IN THE CONTEXT OF PREGNANCY

Trauma has been shown to have particularly deleterious consequences for couple functioning and dyadic communication (Godbout et al., 2013), and, in turn, the couple relationship is important for parenting and child well-being (Gerard et al., 2006; Carlson et al., 2011). Antenatal relationship satisfaction (Cowan and Cowan, 2000; Knauth, 2000) is an important predictor of postpartum relationship satisfaction and parenting, and the transition to parenthood is considered to be a prime time for potential problems to emerge and to be identified (Glade et al., 2005). Results from a number of previous studies indicate that adult attachment states of mind have important implications for couple functioning and relationship quality (Creasey, 2002; Shaver and Mikulincer, 2002; Mikulincer et al., 2013). Similar data are lacking with regard to mentalization and RF, but it has been theorized that RF has direct implications for interpersonal functioning (Fonagy and Target, 1997). Furthermore, for pregnant women with histories of CA&N, RF regarding traumatic interactions with their attachment figures is likely to be especially important for maintaining trust and satisfactory intimate relationships.

### ADAPTATION OF THE RF CODING SYSTEM FOR TRAUMA

The need to develop a system for coding RF about trauma became apparent while coding the AAIs of the pregnant women with CA&N. It became evident that the levels of RF observed in the interviews were generally not maintained when abuse and trauma were discussed. This would, however, not be reflected with the use of a single overall RF score. For this reason, it was decided to obtain a *trauma-specific* RF score. This separate coding of trauma was consistent with the practice for rating attachment, whereby a transcript could be rated for security as well as for the subject being resolved or unresolved regarding trauma. Furthermore, to be sure that we were rating RF regarding trauma reliably, we developed a manual with different levels of RF regarding trauma and asked qualified judges to rate a series of transcripts with regard to trauma-specific RF.

### PRESENT STUDY

In line with the model outlined above, we expected that pregnant women with histories of CA&N would have a lower capacity to think about attachment relationships in mental state terms than that reported for low-risk samples. Furthermore, we expected that pregnant women with CA&N histories would manifest particular difficulties in mentalizing (i.e., RF) in the area of trauma and abuse-related themes (RF-T), as compared to mentalizing about early attachment relationships more generally (RF-G). More specifically, we expected that there would be a significant moderate correlation between RF-G and RF-T, given that they are closely related constructs insofar as they are different dimensions of RF, but, consistent with our expectation that mentalizing difficulties would be more pronounced in the domain of trauma, we hypothesized that the RF-T of these women would be significantly lower than their RF-G. We also hypothesized that difficulties in mentalization regarding trauma, rather than difficulties in mentalization regarding attachment relationships, would be associated with difficulties in investing in the pregnancy, fewer positive feelings about the baby and motherhood, as well as more difficulties in relationships with their partners. In sum, this would provide preliminary evidence of the convergent and discriminant validity of RF-T.

Given the unique opportunity to examine attachment in prospective parents who have all experienced CA&N, a secondary objective of the present study was to provide data on the attachment distributions in the studied group of prospective parents with histories of childhood maltreatment, and to compare the distributions with those found in normative samples and clinical/high-risk abused samples. It was expected that the majority of the pregnant women with CA&N would be insecure with respect to attachment, and unresolved regarding trauma, and that both insecure attachment and unresolved trauma would be significantly more prevalent than reported prevalences in normative samples.

Furthermore, we expected to find evidence of a dose–response relationship between abuse and attachment, with both greater severity of abuse and an increase in the number of abusive experiences being associated with an increased risk of being insecure and unresolved with regard to trauma. However, in line with Fonagy and Luyten’s (2009) proposal that attachment and mentalization are loosely connected, and that individuals may benefit from subsequent experiences that contribute to the development of mentalization, we did not expect to find a similar dose–response relationship between trauma and mentalization.

## MATERIALS AND METHODS

### PARTICIPANTS AND PROCEDURE

Pregnant women were recruited at the obstetrics clinic of a large metropolitan hospital. Prospective participants (*n* = 809) were first screened using the Parental Bonding Instrument (PBI; Parker et al., 1979) to identify women who had experienced lack of parental care in childhood. Exclusion criteria were age below 18, psychotic disorders, acute drug addiction, and living too far outside the city. Of the 131 eligible participants who had scores below the clinical cut-off, 100 women consented to complete the Childhood Experience of Care and Abuse (CECA) interview (Bifulco et al., 1994) to assess histories of trauma. Informed consent for study participation was obtained from all the women. The study was approved by the ethics committee of the Maisonneuve-Rosemont Hospital.

The women ranged in age from 18 to 41 years (*M* = 28.46, SD = 5.58) and more than half (60%) had other children (*M* = 0.81, SD = 0.90). The sample was predominantly French–Canadian (73%) with the remainder being African–American (10%), Hispanic (6%), North African (4%), other Caucasian (4%), Asian (2%), and Native Canadian (1%). Approximately half (52%) of the sample were cohabiting, 34% were married, and 14% were single. In terms of education, 55% had post-secondary education and 41% had been to university. The majority were employed (67%), but approximately half of the sample had annual family income below $30,000, considered to be below the poverty index of approximately $34,000 for a family with one child.

### MEASURES

#### Parental Bonding Instrument

The PBI (Parker et al., 1979) is a 25-item self-report questionnaire developed to assess adults’ perception of the level of parental care and protection/control they received during the first 16 years of childhood. Respondents are questioned about their experiences with each parent separately to obtain care and protection scores for each parent. The psychometric properties of the instrument have been extensively evaluated and it has been shown to have good retest reliability, internal consistency, and validity (Parker, 1989, 1990), with demonstrated stability over twenty years (Murphy et al., 2010). In the present study, the PBI was used as a screening instrument to identify pregnant women who had experienced low parental care from both parents in childhood, using standard PBI cut-off scores (care scores of 27 for maternal figure and 24 for paternal figure).

#### Childhood Experience of Care and Abuse

The CECA (Bifulco et al., 1994) is a semi-structured interview designed to retrospectively measure adverse childhood experiences. The CECA assesses different domains of childhood experiences including antipathy from parents, neglect, physical abuse, and sexual abuse. Ratings are made for each domain on a 1–4 scale (4 *little/none*, 3 *some*, 2 *moderate*, 1 *marked*) based on a rating manual that provides explicit examples of the type of parental behavior that is considered to represent different levels of severity. The investigator-based format of the CECA has the advantage of not depending on participants to have previously categorized their own childhood experiences as abusive. The CECA has been demonstrated to have good psychometric properties including good inter-rater reliability and also validity as determined by agreement between sisters’ independent accounts (Bifulco et al., 1994).

All CECA interviews were audiotaped and subsequently coded by trained raters. We classified the women into two groups; an *abused* group comprising women who had been exposed to physical or sexual abuse in addition to neglect or antipathy, and a *neglect* group in which we included individuals with exposure to neglect and antipathy without physical or sexual abuse. Given how little previous research has examined the relationship between maltreatment, attachment and RF, we chose to examine the impact of abuse also including women who had experienced *some* maltreatment (severity rating of 3).

#### Adult Attachment Interview (AAI)

The AAI is a semi-structured interview designed to assess adults’ state of mind with respect to their attachment relationships with their parents during childhood (George et al., 1985). Based on their general strategy evident in discussing attachment relationships, participants are categorized as *secure-autonomous* (F), *insecure-dismissing* (Ds), *insecure-preoccupied* (E), or *cannot classify* (CC), using Main et al.’s (2002) coding manual. According to the manual, secure-autonomous individuals provide relatively clear, coherent, and succinct responses and accounts that are consistent. Individuals with challenging backgrounds can also be classified as *secure-autonomous* if they provide coherent accounts of their experiences. *Insecure-dismissing* participants, by contrast, provide highly positive and idealized accounts of their parents that are contradicted later in the interviews, and insist that they are unable to remember experiences with their attachment figures. For *insecure-preoccupied* individuals, the interview questions appear to provoke excessive activation of attachment-related memories and confused, angry, or passive preoccupation with attachment figures. They provide long, rambling, and confusing descriptions and appear to lose the capacity to focus their discourse and respect the rules of communication such as providing clear and succinct accounts. Both dismissing and preoccupied individuals are considered to be insecure. In rare cases, when no single strategy is predominant, the *cannot classify* category is used.

***Unresolved classification***. Participants who have experienced loss or traumatic experiences are assessed to determine whether they are unresolved/disorganized (U/d). Lack of resolution is coded on a scale (1–9) with scores of 5 and higher considered to reflect lack of resolution (Main et al., 2002). Lack of resolution manifests in lapses in the monitoring of reasoning or discourse when individuals discuss traumatic experiences. Abuse is considered to include “any form of experiences which have been overwhelmingly frightening and heightening of fearful attention toward prospective incidents” (Main et al., 2002, p. 137). Current convention is to combine the U/d and CC classifications because of potential commonalities in etiology and sequelae (Bakermans-Kranenburg and van IJzendoorn, 2009), a convention that was followed in the present study.

The AAI has good psychometric properties, as evidenced in high test–retest reliability, validity, and stability over time (Bakermans-Kranenburg and van IJzendoorn, 1993; Benoit and Parker, 1994; Sagi et al., 1994). In the present study, transcripts were coded by a rater who was trained to be reliable to the coding standards of the Berkeley laboratory of Mary Main and Erik Hesse. The coder was blind to abuse and neglect as assessed by the CECA, to the goal of the study, and to the composition of the sample.

#### Reflective functioning

The AAI was coded for RF, using the RF manual (Fonagy et al., 1998). The psychometric properties detailed in the manual include high inter-rater reliability, as well as good discriminant, divergent, and predictive validity across a number of samples. RF is scored on a scale of –1 to 9, with every score representing a different level of mental state explanation. Ratings of –1 indicate an attack on mentalization. Ratings of 0 indicate refusal to engage in mentalization. Ratings of 1 indicate the absence of any recognition of mental states, so that interpersonal reactions are described only in behavioral terms, or individuals only in terms of global personality traits. Ratings of 3 indicate a limited capacity to acknowledge mental states but without an understanding of how mental states function. Ratings of 5 indicate a basic capacity for RF and a basic understanding of how mental states interact and influence behavior. Higher ratings indicate increasingly sophisticated and full mental state accounts of interactions and reactions, with ratings of 9 indicating exceptional mental state thinking and insights. RF is typically scored based on all AAI questions that explicitly demand an appreciation of mental states (e.g., “why did your parents behave as they did during your childhood?”). An overall RF score that represents the respondent’s characteristic level of RF is derived based on the individual scores, using a decision algorithm outlined in the manual. This takes into account the respondent’s most frequent level of RF responses as well as the frequency of responses characterized by high and low RF. The RF coding system has been demonstrated to have good psychometric properties (Fonagy et al., 1998; Taubner et al., 2013). In the present study, RF was rated by two qualified raters who were trained by the developers of the RF coding system and have more than 10 years of experience in rating RF of adults, parents and children. The inter-rater reliability was computed on the 10 cases (10% of the total sample) considered to be the most difficult. Intraclass correlations of 0.79 obtained for the RF ratings of these 10 cases suggested good reliability even with challenging transcripts. RF raters were blind to CECA scores.

***Reflective functioning regarding traumatic experiences***. In addition to obtaining the overall RF score as outlined above, which relates to attachment relationships more generally and which we will term RF-G, we were particularly interested in RF regarding traumatic experiences (RF-T). To obtain an RF-T score that would serve as a good indicator of RF specifically about trauma, we used the RF scale (range –1 to 9) to rate all passages during which abuse was directly probed or explicitly discussed. The structure of the AAI permits such separate ratings, given that when respondents have experienced childhood abuse, the questions eliciting mentalization about this abuse are asked on two distinct occasions during the course of the interview: first when explicitly discussing the abuse and then again toward the end of the interview when discussing the overall history, when the interviewer asks, for example, “*Do you feel the experience of having been physically abused by your father affects you now as an adult*?” (Essentially the questions that are used to rate RF-T are exactly the same as the questions used to assess unresolved trauma in the attachment coding system). Ratings of other demand questions such as “*In general, how do you think your overall experiences with your parents have affected your adult personality?*” were used to obtain an RF-G score. Given that the AAI provides a set of questions regarding childhood abuse, but no such detailed questions regarding childhood neglect or other potentially traumatic incidents, only childhood abuse can be coded for RF-T. This means that where a respondent manifested low mentalization only when discussing abuse experiences this would be captured in the RF-T score and would not lower the global RF-G score. This is consistent with the approach used when coding attachment: participants who manifest evident lapses in the monitoring of discourse specifically when discussing loss or traumatic experiences can still be considered to be globally coherent and be classified as *secure-autonomous*, while also having an *unresolved* classification (Main et al., 2002).

In order to facilitate reliable rating of RF-T, we developed an addendum to the existing RF coding manual, specifically elaborated to provide examples of the different types and levels of RF-T. All examples of mentalization regarding trauma were extracted from the AAIs and rated by two raters. When there were divergences in rating this was discussed and a consensus rating was reached in consultation with a third, expert RF rater. The rationale for each rating was elaborated and clearly formulated, so that this could be used (and subsequently clarified) when similar examples were encountered.

To verify whether our RF-T addendum to the RF coding manual could be used to train raters to become reliable in coding RF-T, we subsequently used the RF-T addendum to train four postgraduate students working in our laboratory, who were already experienced in coding RF in other studies. The training comprised an introduction to the RF-T manual, discussion of the different levels of RF-T and examples, and additional individual practice ratings followed by discussion. After 9 h of training, excellent agreement between raters was achieved. The four raters then rated 20 protocols, and the intraclass correlation between the four raters was 0.87, suggesting excellent reliability.

#### Contextual Assessment of Maternity Experience (CAME)

The Contextual Assessment of Maternity Experience (CAME; Bernazzani et al., 2005) interview was developed to assess the psychosocial context relevant to a pregnant woman’s experience during the transition to motherhood, including her perception of the quality of her relationship with her partner, maternal commitment, and her feelings toward pregnancy, motherhood, and the baby. In the present study the CAME was used to evaluate the abovementioned factors. Five dimensions of couple functioning and maternal commitment and feelings toward the pregnancy, motherhood, and the baby are assessed, and each dimension of functioning is rated on a scale of 1–4 (4 *little/none*, 3 *some*, 2 *moderate*, 1 *marked*). Interviews were recorded and were subsequently coded by trained raters following a rating manual. The CAME has been demonstrated to have good concurrent and predictive validity and the dimensions have been shown to have good internal consistencies in different samples (Bernazzani et al., 2005).

### STATISTICAL ANALYSES

With regard to RF, a Pearson correlation was used to evaluate the association between RF-G and RF-T, and a paired samples *t*-test was used to compare RF-G and RF-T scores in the same participants. Linear regressions were performed to evaluate the association between the dose of maltreatment and levels of RF-G and RF-T. Next, *t*-tests were used to assess whether RF-G and RF-T were related to attachment classification. Finally, multiple linear regressions were performed to evaluate the best predictors of parenting and couple functioning among RF-G, RF-T, unresolved versus non-unresolved attachment states of mind, and secure versus insecure attachment states of mind.

*Z*-tests were used to compare the attachment distributions of the women with CA&N recruited from the community in this study with attachment distributions reported for normative and clinical/high-risk abused samples. Chi-square analysis and *z*-tests were also used to compare the attachment distributions of women who had experienced severe versus less severe abuse. Linear regressions were performed to evaluate the association between the dose of maltreatment and unresolved attachment classification.

## RESULTS

### CHARACTERISTICS OF CHILDHOOD ABUSE AND NEGLECT

All the women reported some type of maltreatment in the CECA interview. Types of CA&N experienced by study participants are summarized in **Table 1**. Abuse and neglect were consistently reported across the AAI and the CECA interviews for 88% of participants, with the CECA providing a more comprehensive assessment of maltreatment. Of the 12 women who described abusive childhood experiences during the CECA interview but did not report abuse during the AAI, five reported some physical abuse from biological parents and seven reported extra-familial sexual abuse.

**Table 1:** Type and severity of childhood abuse and neglect experienced by the pregnant women.

	Cases		Severity			Primary perpetrator							
	*N* = 97		Some	Moderate	Severe	Biological parent	Alternative caregiver	Sibling	Relative or other
Physical abuse	56		23 (41%)	24 (43%)	9 (16%)	42 (75%)	6 (11%)		8 (14%)
Sexual abuse	39		11 (28%)	11 (28%)	17 (44%)	4 (10%)	11 (28%)	4 (10%)	20 (51%)
Neglect	76		26 (34%)	28 (37%)	22 (29%)	75 (99%)	1 (1%)		
Antipathy	77		26 (34%)	32 (42%)	19 (25%)	77 (100%)			

*Childhood Experience of Care and Abuse (CECA) data were missing for three cases*.

### REFLECTIVE FUNCTIONING

The RF-G scores were normally distributed, with a mean around 4 (*M* = 4.14, SD = 1.95). Eighteen percent of the participants had RF-G scores ranging from –1 to 2, 38% had scores of 3 or 4, 31% had scores of 5 or 6, and 13% had scores between 7 and 9. RF-T scores were also normally distributed, with a mean of 2.78 (SD = 1.96). Fifty percent of participants had RF-T scores ranging from –1 to 2, 28% had scores of 3 or 4, 17% had scores of 5 or 6 and 5% had scores between 7 and 9. Although RF-T scores and RF-G scores were significantly correlated (*r* = 0.61, *p* < 0.001), mean RF-T was significantly lower, as confirmed by a paired *t*-test [*t*(63) = 4.93, *p* < 0.001, *d* = 0.70]. There was no association between the dose of maltreatment and RF-T [β = 0.07, *t*(35) = 0.48, *p* = 0.64] or RF-G [β = -0.04, *t*(55) = -0.43, *p* = 0.67] in the women in this sample.

### REFLECTIVE FUNCTION, INVESTMENT IN PREGNANCY, AND QUALITY OF RELATIONSHIP WITH PARTNER

Multiple regression analyses were used to identify the best predictors of engagement in pregnancy and quality of couple functioning (**Table 2**). RF-T, RF-G, unresolved versus non-unresolved attachment, and secure versus insecure attachment were entered simultaneously. RF-T was the best single predictor of engagement in the pregnancy (β = –0.42; *p* = 0.01), positive feelings regarding the pregnancy (β = –0.37; *p* = 0.03), sense of commitment toward maternity (β = –0.35; *p* = 0.03), and overall quality of relationship with partner (β = –0.57; *p* < 0.001).

**Table 2:** Regression analyses examining the contribution of RF-G, RF-T, secure vs. insecure attachment, and organized vs. unresolved attachment to predicting parenting and quality of relationship with partner in pregnant women with histories of physical or sexual abuse.

	Engagement in pregnancy			Positive affects toward pregnancy			Engagement in maternity			Overall quality of relationship with partner		
	β	*t*	*p* value	β	*t*	*p* value	β	*t*	*p* value	β	*t*	*p* value
**RF-G**	0.30	1.78	0.08	0.13	0.74	0.46	0.17	1.03	0.31	0.20	1.31	0.20
**RF-T**	–0.42	–2.66	0.01**	–0.37	–2.20	0.03*	–0.35	-2.30	0.03*	–0.57	–3.84	<0.001***
**Secure vs. insecure**	0.26	1.27	0.21	–0.04	–0.21	0.83	0.34	1.84	0.07	0.30	1.67	0.10
**Organized vs. unresolved**	–0.07	–0.37	0.71	0.01	0.06	0.95	–0.32	-1.95	0.06	0.03	0.20	0.84

*p ≤ 0.05, **p ≤ 0.01, ***p ≤ 0.01.

### ATTACHMENT DISTRIBUTIONS AND THEIR CORRELATES

In our sample, 37% of women with CA&N had secure attachment states of mind, 21% were dismissing, 5% were preoccupied and 37% were unresolved. The majority of unresolved participants were unresolved regarding abuse (91%) rather than loss (9%). Attachment distributions are presented in **Table 3** and contrasted with attachment data from Bakermans-Kranenburg and van IJzendoorn’s (2009) meta-analysis. Results from *z*-tests for two independent proportions showed that pregnant women with CA&N in this sample were characterized by a significantly lower percentage of secure attachment (*z* = 3.46, *p* < 0.01) and a significantly higher percentage of unresolved attachment (*z* = 4.28, *p* < 0.01) than proportions reported in normative samples. Compared to attachment distributions reported for abuse samples recruited in clinical or high-risk contexts, our community sample had, as expected, a significantly higher proportion of secure attachment (*z* = 4.71, *p* < 0.01) and significantly less unresolved attachment (*z* = 10.95, *p* < 0.01).

**Table 3:** Attachment classifications of the total sample, abuse and neglect subgroups and comparison groups.

		State of mind with regard to attachment			
	*N*	Secure 4-ways (3-ways)	Dismissing 4-ways (3-ways)	Preoccupied 4-ways (3-ways)	Unresolved 4-ways


Total sample	100	37% (44%)	21% (28%)	5% (28%)	37%
Neglect subgroup	26	46% (46%)	35% (35%)	15% (19%)	4%
Abuse subgroup	74	34% (43%)	16% (26%)	1% (31%)	49%
Normative mothers^a^	748	56% (58%)	16% (23%)	9% (19%)	18%
Abuse/PTSD^a^	271	14% (43%)	11% (20%)	7% (37%)	68%

*^a^Reported by Bakermans-Kranenburg and van IJzendoorn (2009)*.

Next, we compared attachment distributions of the women in our sample with histories of severe abuse with those who had experienced less severe abuse, using the severe versus non-severe dichotomization of the CECA. Severely abused women differed significantly from those who had experienced less severe abuse in terms of attachment classification (secure, insecure, unresolved; *χ*^2^ = 21.83, *p* < 0.001) and were significantly more likely to be unresolved (*z* = 4.47, *p* < 0.01) and to have insecure attachment states of mind (*z* = 2.30, *p* < 0.05).

Furthermore, the results of logistic regression indicate that the number of different types of maltreatment to which a mother had been exposed during childhood was associated with an increased risk of being classified as unresolved (β = 1.14, Wald = 9.59, *p* < 0.01, OR = 3.13).

### ATTACHMENT AND REFLECTIVE FUNCTIONING

The RF-G was related to attachment classifications: women with secure attachment states of mind (*M* = 5.16, SD = 1.69) displayed higher RF-G than women with insecure states of mind (*M* = 3.63, SD = 1.96), [*t*(98) = 3.58, *p* = 0.001, *d* = 0.84] (see **Table 4**). However, there were no differences in RF-G between women with organized attachment classifications (*M* = 4.30, SD = 1.88) and those classified as unresolved (*M* = 3.86, SD = 2.06), [*t*(98) = 1.08, *p* = 0.28].

No association was found between RF-T and attachment: women with histories of abuse who were not unresolved regarding trauma (*M* = 2.97, SD = 1.92) did not have significantly higher RF-T than those who were unresolved (*M* = 2.63, SD = 2.02), [*t*(62) = 0.68, *p* = 0.50]. There was also no significant difference between the RF-T of women with secure (*M* = 3.20, SD = 1.94) and insecure (*M* = 2.59, SD = 1.97) states of mind [*t*(62) = 1.15, *p* = 0.25].

**Table 4:** Reflective functioning (RF-G) and attachment classifications.

Attachment	*N*	*M*	*SD*
Secure	43	5.16	1.69
Dismissing	25	3.08	1.91
Preoccupied	21	3.81	1.78
Cannot classify	11	3.18	1.40
Total	100	4.14	1.95

Significant *post hoc* tests: Secure vs. dismissing, *p* < 0.000; Secure vs. preoccupied, *p* = 0.02; Secure vs. cannot classify, *p* = 0.006; *p < 0.05, **p < 0.01.

## DISCUSSION

The aim of this study was to investigate mentalization and attachment in pregnant women with histories of CA&N and to examine whether these women manifest general deficits in mentalization about attachment relationships compared with the norms reported for low-risk samples. Furthermore, we expected that women with CA&N histories would manifest significant deficits in mentalization about traumatic experiences compared to mentalization about attachment relationships. We were particularly interested in determining whether RF-T, as measured using the coding manual specially elaborated for this purpose, is a valid construct. More specifically, we expected that RF-T would be related to RF-G, but that only RF-T would be associated with pregnant women’s self-reported commitment to and feelings about pregnancy, motherhood, and the baby, as well as the quality of their relationships with their partners.

The mean RF-G of women in this sample (4.14) was somewhat lower than the mean (5.00) reported in low-risk populations. This is consistent with developmental research showing deficits in capacities broadly overlapping with mentalization, such as children’s self-representation, theory of mind, and emotional understanding associated with abuse and neglect. However, their capacity to mentalize about early attachment relationships was nonetheless greater than expected, and we did not observe a global deficit in mentalization. The pregnant women in this sample demonstrated a propensity, albeit incomplete, to use a mental state understanding in their accounts of attachment relationships. This raises questions regarding the pathways through which adults who have been abused as children acquire mental state thinking and suggests that some individuals find opportunities, possibly outside the family, to develop a mental state understanding as a way of surviving challenging emotional experiences. An alternative explanation is that they are compelled to elaborate an account of their difficult childhood experiences.

The most important findings of this study concern the marked deficit in mentalization regarding trauma observed in the pregnant women with CA&N histories, as assessed using our RF-T coding manual. Compared with their RF regarding attachment relationships, women with CA&N histories manifested striking difficulties in mentalization regarding trauma, with RF-T being significantly lower than RF-G. These findings require replication, but suggest that individuals with CA&N histories have been able to develop accounts of their difficult early attachment relationships in mental state terms, but find it particularly difficult to develop adequate mental state accounts of abusive experiences. The findings add to, but are broadly consistent with, extensive research evidence of avoidance and dissociation of traumatic memories and difficulties in accessing and putting into words memories that have been stored as experiences rather than as narratives (Brewin, 2011; Allen, 2013).

In order to examine evidence of the validity of the RF-T construct, we examined whether RF had a unique contribution in the prediction of functioning in two key areas, namely, investment in the pregnancy and quality of relationship with the partner. The findings provide further evidence suggesting that RF-T is indeed a valid construct and can be meaningfully differentiated from RF-G. RF-T was the best and only predictor of women’s engagement in pregnancy, positive feelings toward the pregnancy and engagement in motherhood, as well as the quality of their relationship with their partner. While for most prospective parents pregnancy provokes mixed feelings, including both joy and apprehension, those with a history of CA&N are very likely to face additional anxieties and threat. Dissociation may have helped them to maintain functioning, but will no longer be adequate when they are faced with the major challenge of becoming a parent. For prospective parents with CA&N histories the inevitable anxiety around being responsible for a new life can easily become overwhelming in the context of unmentalized trauma. Furthermore, at a theoretical level, the proposition is frequently made that mentalization has important implications for the quality of interpersonal relationships, but to our knowledge this is the first direct evidence to support this theory. In addition, the relationship between RF-T and the quality of interpersonal relationships was strong, and showed that difficulties in thinking about past traumatic experiences were associated with poor relationship quality. Linking our findings to the dual processing model proposed by Fonagy and Luyten (2009), it may be that RF-T or mentalization of traumatic experiences helps women with CA&N histories to maintain *reflectiveness* for longer at times of stress when trauma-related affects are triggered in the context of attachment relationships, before switching to more automatic *reflexive* modes of mentalizing that are highly influenced by their traumatic experiences.

Given the unique opportunity of gaining attachment data on a relatively large number of pregnant women, all with histories of abuse and neglect, and the absence of data on the attachment of adults with CA&N histories in the community, we were also interested in the pregnant women’s state of mind with respect to attachment. The study findings show that the majority of pregnant women with CA&N histories had insecure attachment states of mind, but that one-third of women with CA&N had secure attachment states of mind. These findings are consistent with those of Stovall-McClough et al. (2008), who also found that a similar proportion of women with histories of CA&N had secure attachment states of mind, and provides further evidence that individuals with CA&N do not necessarily have insecure states of mind. This is likely to remain masked when participants are recruited in clinical or high-risk populations. Of the women who had experienced *abuse*, approximately half were unresolved with regard to trauma, indicating that they showed cognitive lapses in the monitoring of reasoning or discourse when asked about traumatic experiences and their impact, suggesting that traumatic memories and associated affects overwhelm their capacity to maintain cognitive integration when addressing traumatic themes.

Furthermore, the findings indicate that severely abused women are more likely to be unresolved and to have insecure attachment states of mind, and that women who had been exposed to a higher number of traumatic experiences are more likely to be unresolved than women whose experience of abuse were less severe or less frequent. This adds to the evidence that there is a dose–response relationship between early adversity and unresolved trauma, in terms of both the severity of trauma individuals have been exposed to and the number of traumatic experiences.

In line with the theory that mentalization develops in the context of attachment relationships (Fonagy and Target, 1997, 2006), women with secure attachment states of mind had significantly higher RF-G. However, there was no association between being unresolved with regard to trauma and mentalization regarding trauma (measured as RF-T). This suggests that RF-T assesses a distinct but complementary dimension of trauma processing. Furthermore, the dose and severity of abuse were linked to the risk of being unresolved with regard to trauma, but not to RF-T. This would seem to suggest that how an individual mentalizes about trauma is not determined by the actual traumatic experience.

The findings of the present study can be seen to be in line with, and to extend, Fonagy’s (1993) conceptualization of the “ghosts in the nursery” as an *absence*. The findings advance our understanding with regard to the specific nature of this absence of mentalization and suggest that it is not simply an absence of mentalization about early attachment relationships, but more specifically the *absence of mentalization about trauma* that may be most important. The findings of this study showed that RF-T was linked to adaptive functioning in two important areas, including the women’s commitment to pregnancy and positive feelings about the baby and motherhood, as well as the quality of the relationship with their partner. We propose that in contexts where trauma-related affects are triggered, RF-T may be important for maintaining appropriate interpersonal functioning and self-regulation of helplessness, fear, hostility, and aggression. This is likely to be especially important during interactions with infants and children, where displays of aggression and hostility by mothers are particularly inappropriate and harmful.

In line with Fraiberg et al.’s (1975) observation that being prepared to remember the pain of past trauma distinguishes those who make a conscious effort not to repeat trauma and is a central factor that protects them from repeating the trauma, we hypothesize that the absence or failures of mentalization regarding trauma is likely to play a key role in the trans-generational transmission of trauma. Findings from recent microanalytic studies of parent-infant interaction by Beebe et al. (2010) highlight the importance of inappropriate, unexpected responding at 4 months as the primary determinant of subsequent disorganized attachment. From a psychoanalytic perspective, Bion’s (1962) model of an absence of containment may be helpful to understand how such relatively minor absences may have devastating consequences in contexts where the infant’s behavior triggers trauma related ideation in the caregiver. As the infant’s distress is the most probable trigger for such brief but dramatic failures of mentalization with the activation of the mother’s memories of trauma, it is indeed these experiences in the infant which will remain without adequate second order representation that we believe is created from the mother’s mirroring response. We have assumed that the absence of the contingent marked mirroring response, the product of the mother’s reflection of her empathic response, creates an unmentalized alien core around the child’s experience. We have suggested that Winnicott’s (1967) model of the failure of maternal mirroring may be operationalized as the absence of marked contingent mirroring and results in the infant’s internalization of the mother’s re-experience of un-mentalized trauma. This may be a seed for the experience that we have termed the “alien self,” by which we mean a direct experience of distress which is both felt to be within the self and yet also feels inexplicably to be occurring independent of other aspects of subjectivity. For example, the feeling of badness that cannot be mitigated by reassurance and exists despite a generally benign environmental context may be exactly such an internalization of temporary maternal absence. Clinically when patients re-experience these moments, they tend to become closest to feelings of de-realization and dissociation. These experiences of un-metabolized distress can lead to experiences of incoherence of the self that are so painful that they are only met through complex processes of projective identification where alien parts of the self are lodged in another, which relieves the incoherence, but creates an alternative problem of provoking the other to become the disowned self fragment that the patient requires the other to be. In this way a persecutory self-experience can be actualized as a persecution and the internal sense of feeling tortured is exchanged for a feeling of being victimized. This, we believe, is a relatively economic model to explain the trans-generational transmission of the experience of trauma, and should be tested through studies with data on mother-infant interaction and infant attachment. More specifically, further clarification of the dynamic relationships between mentalization and defenses, and the association with maternal interactional behavior and interpretation of infant emotion, is required.

Although this study has several strengths, including the use of state-of-the-art measures of adult attachment, RF, and maltreatment, the findings need to be interpreted in the context of some limitations. While we went to considerable lengths to ensure that RF-T was reliably coded, and elaborated an RF-T coding manual especially for this purpose, the findings require replication, and demonstration of the reliability of the RF-T coding manual needs to be repeated. The finding that RF-T was related to functioning in two different domains suggests that it is a valid indicator of mentalization regarding trauma with potentially good predictive value. The best test of the validity of the RF-T construct will be whether it predicts infant attachment disorganization, and whether it explains more variance in infant attachment disorganization than unresolved trauma does. Further research is needed to clarify the relationship between RF-T, unresolved trauma, dissociation, defenses, and PTSD and their role in pregnant women’s adaptation to the present and past traumatic experiences.

## CONCLUSION

The most important new finding of this study is that prospective mothers with histories of CA&N show marked deficits in mentalizing in the specific area of *trauma.* Difficulties in mentalizing about abusive experiences is likely to leave adults with CA&N histories with an area of vulnerability, and the absence of mentalization about events that are likely to have triggered intense affects such as fear, helplessness or anger can be considered to increase the risk of dysregulation in challenging interpersonal contexts, and can also be considered to make the transition to parenthood more conflicted. These important deficits in mentalization specifically associated with trauma would not have been evident had we examined only RF-G. In sum, the findings of this study suggest that the RF-T coding manual and scale are an important contribution that can facilitate the reliable rating of RF-T. Future research is needed to clarify the implications of RF-T for infant attachment disorganization.

## Conflict of Interest Statement

The authors declare that the research was conducted in the absence of any commercial or financial relationships that could be construed as a potential conflict of interest.
